# School electrocardiography screening program prompts the detection of otherwise unrecognized atrial septal defect in children in Japan

**DOI:** 10.3389/fped.2024.1396853

**Published:** 2024-06-03

**Authors:** Noriko Yodoya, Hirofumi Sawada, Yoshihide Mitani, Hiroyuki Ohashi, Naoki Tsuboya, Kazunobu Ohya, Mami Takeoka, Hidetoshi Hayakawa, Masahiro Hirayama

**Affiliations:** Department of Pediatrics, Mie University Graduate School of Medicine, Mie, Japan

**Keywords:** pediatrics, ASD, electrocardiography, mass screening, school health program

## Abstract

**Background:**

Atrial septal defect (ASD) is a congenital heart disease that often presents without symptoms or murmurs. If left untreated, children with ASD can develop comorbidities in adulthood. In Japan, school electrocardiography (ECG) screening has been implemented for all 1st, 7th, and 10th graders. However, the impact of this program in detecting children with ASD is unknown.

**Methods:**

This is a retrospective study that analyzed consecutive patients with ASD who underwent catheterization for surgical or catheter closure at ≤18 years of age during 2009–2019 at a tertiary referral center in Japan.

**Results:**

Of the overall 116 patients with ASD (median age: 3.0 years of age at diagnosis and 8.9 years at catheterization), 43 (37%) were prompted by the ECG screening (Screening group), while the remaining 73 (63%) were by other findings (Non-screening group). Of the 49 patients diagnosed at ≥6 years of age, 43 (88%) were prompted by the ECG screening, with the 3 corresponding peaks of the number of patients at diagnosis. Compared with the non-screening group, the screening group exhibited similar levels of hemodynamic parameters but had a lower proportion of audible heart murmur, which were mainly prompted by the health care and health checkups in infancy or preschool period. Patients positive for a composite parameter (rsR' type of iRBBB, inverted T in V4, or ST depression in the aVF lead) accounted for 79% of the screening group at catheterization, each of which was correlated with hemodynamic parameters in the overall patients.

**Conclusions:**

The present study shows that school ECG screening detects otherwise unrecognized ASD, which prompted the diagnosis of the majority of patients at school age and >one-third of overall patients in Japan. These findings suggest that ECG screening program could be an effective strategy for detecting hemodynamically significant ASD in students, who are asymptomatic and murmurless.

## Introduction

Atrial septal defect (ASD) is a common congenital heart disease with an estimated incidence of 56 per 100,000 live births (1). Children with isolated ASD are generally asymptomatic and frequently do not present with significant heart murmurs (1–3). As a result, diagnosis or referral for ASD is occasionally delayed until adulthood, when patients may present with comorbidities such as arrhythmias, heart failure, and pulmonary hypertension (4–6). Early shunt closure is safe and effective, and studies have shown that performing the procedure before the age of 25 provides a normal life expectancy (7). Therefore, it is important to develop a strategy for detecting asymptomatic ASD patients during childhood.

The 12-lead electrocardiogram (ECG) is a simple and inexpensive tool commonly used in the cardiology clinic. ECG findings in ASD include right bundle branch block (iRBBB), rsR' type of iRBBB, right axis deviation, isolated negative T waves, and notches in the lower leads (II, III, aVF) (3, 8–10). In Japan, school ECG mass screening for cardiovascular diseases has been conducted since 1995, when this system was legislated (8). With the use of the guidelines for systematically interpreting ECG, this system has an impact on the early detection of substrates for sudden cardiac death, long QT syndrome (11), myocardial diseases (12) and idiopathic pulmonary arterial hypertension (13) at the community level. Although ASD cases are occasionally found by school ECG screening (14, 15), the impact of this healthcare system on detecting ASD in childhood has not been investigated.

In the present study, we investigated the impact of school ECG-based screening system in detecting children with hemodynamically significant ASD at a tertiary referral center in Japan.

## Methods

### Study design

This is a retrospective observational study at a single center, in which consecutive patients with isolated ASD at the age of 18 years or younger during 2009–2019 at Mie University Hospital, Japan were investigated. The study was approved by the Institutional Review Board at Mie University Graduate School of Medicine (No. H2019-041) in accordance with the guidelines for epidemiological research issued by the Ministry of Health, Labor and Welfare of Japan.

### Study setting

Mie University Hospital is the only tertiary referral center for Pediatric Cardiology in a tertiary medical service area Mie prefecture, Japan, covering a population of 310,000 persons at the age of 18 years or younger. All patients for surgical or transcatheter closure of ASD underwent cardiac catheterization and angiography in this hospital during the study period.

In Japan, government-sponsored health checkup has been performed for infants and preschool children at the age of 1, 4, 7, 10, 18 months, 3 years, and 5 years. Each checkup includes history taking for the developmental, mental and nutritional surveillance and comprehensive physical examination including chest auscultation but not ECG. School ECG-based mass screening (ECG-screening) program has been implemented for all the first graders in elementary (1st grade, 6–7 years of age), middle (7th grade, 12–13 years), and high schools (10th grade, 15–16 years). In some districts, additional ECG-screening has been done at 4th grade (9–10 years of age) in elementary schools. The primary screening in this program includes an interview sheet filled out by the guardian, physical examination including chest auscultation by the school physician and ECG which is recorded at the school and assessed by the regional committees organized by internists, pediatricians and pediatric cardiologists. Subsequently, students who meet the criteria in guidelines established by the Japanese Circulation Society (JCS) and the Japanese Society of Pediatric Cardiology and Cardiac Surgery (JSPCCS) are requested to visit local hospitals for the secondary screening in which chest x-ray, echocardiography or exercise ECG were performed under the health care coverage. Subjects who have abnormal findings in the secondary screening at the local hospitals are finally referred to the tertiary referral hospitals for the final diagnostic examination and treatment (Table 1).

**Table 1:** Child hearth care system in Japan.

Health checkup for infants and preschool children
Time of implementation Ages 1, 4, 7, 10, 18 months, 3, 5 years old
Procedures 1. Checkups at a pediatrician's office or local health center Available information • Interview sheet filled out by parents • Growth chart • Physical examination by a pediatrician 2. Referral to secondary or tertiary hospital • Refer subjects with possible medical problem
ECG-based heart disease screening at schools
Time of implementation • 1st, 7th and 10th grade • Additional screening at 4th grade in some districts.
Procedures 1. Primary screening at schools Available information • Interview sheet filled out by parents • Physical examination by school physician • Electrocardiography 2. Secondary screening at clinics or hospitals Available tests • Electrocardiography • Echocardiography • Exercise stress tests 3. Final diagnosis and treatment at tertiary centers

The infant or preschool checkup include history taking for the developmental, mental and nutritional surveillance and comprehensive physical examination including chest auscultation but not electrocardiography (ECG). The primary screening in the ECG-based heart disease screening includes an interview sheet, chest auscultation and ECG. ECGs are assessed by the regional committees organized by internists, pediatricians and pediatric cardiologists. Students who meet the criteria in guidelines established by the Japanese Circulation Society and the Japanese Society of Pediatric Cardiology and Cardiac Surgery are requested to visit local hospitals for the secondary screening. Subjects who have abnormal findings in the secondary screening at the local hospitals are finally referred to the tertiary referral hospitals for the final diagnostic examination and treatment.

ECG, electrocardiography.

### Data collection

The patient database for cardiac catheterization and angiography in the Department of Pediatrics at Mie University Hospital, which collected patient data prospectively, was searched for individuals with ASD. Consecutive patients with isolated ASD were recruited: patients with sinus venosus ASD with partial anomalous pulmonary venous connection and patients with genetic or syndromic disorders, such as Down syndrome, were included. Patients with other intracardiac diseases, including pulmonary valve stenosis with pressure gradient between main pulmonary artery and right ventricle ≥15 mmHg by catheterization, were excluded. Data collection was completed by the end of March 2022.

The data collected from the database included the sex and age of the patients, time of diagnosis of ASD, mode of detection of ASD, symptoms at diagnosis, coexisting diseases, ECG findings, including right axis deviation (≥+120°), incomplete right bundle branch block (iRBBB) (defined as QRS width <0.12 s in middle and high school and <0.10 s in lower elementary school in the right precordial lead V1 or V2) and rsR' type of iRBBB (R’ > R in V1 or V2 and R’V1 ≥ |SV1|), T-wave inversion in V4 (≥0.1 mV), ST depression (≥0.05 mV, and ST segment is horizontal or downslope) in aVF, the crochetage pattern, which is a notch near the apex of the R wave in the lower leads (II, III, aVF) and right heart catheterization data at examination. The 12-lead electrocardiogram of each patient was evaluated using the criteria in the guideline established by JCS and JSPCCS (8) (Supplementary Table S1), and all ECG abnormalities noted at diagnosis were confirmed by three board-certified pediatric cardiologists.

**Figure 1:**
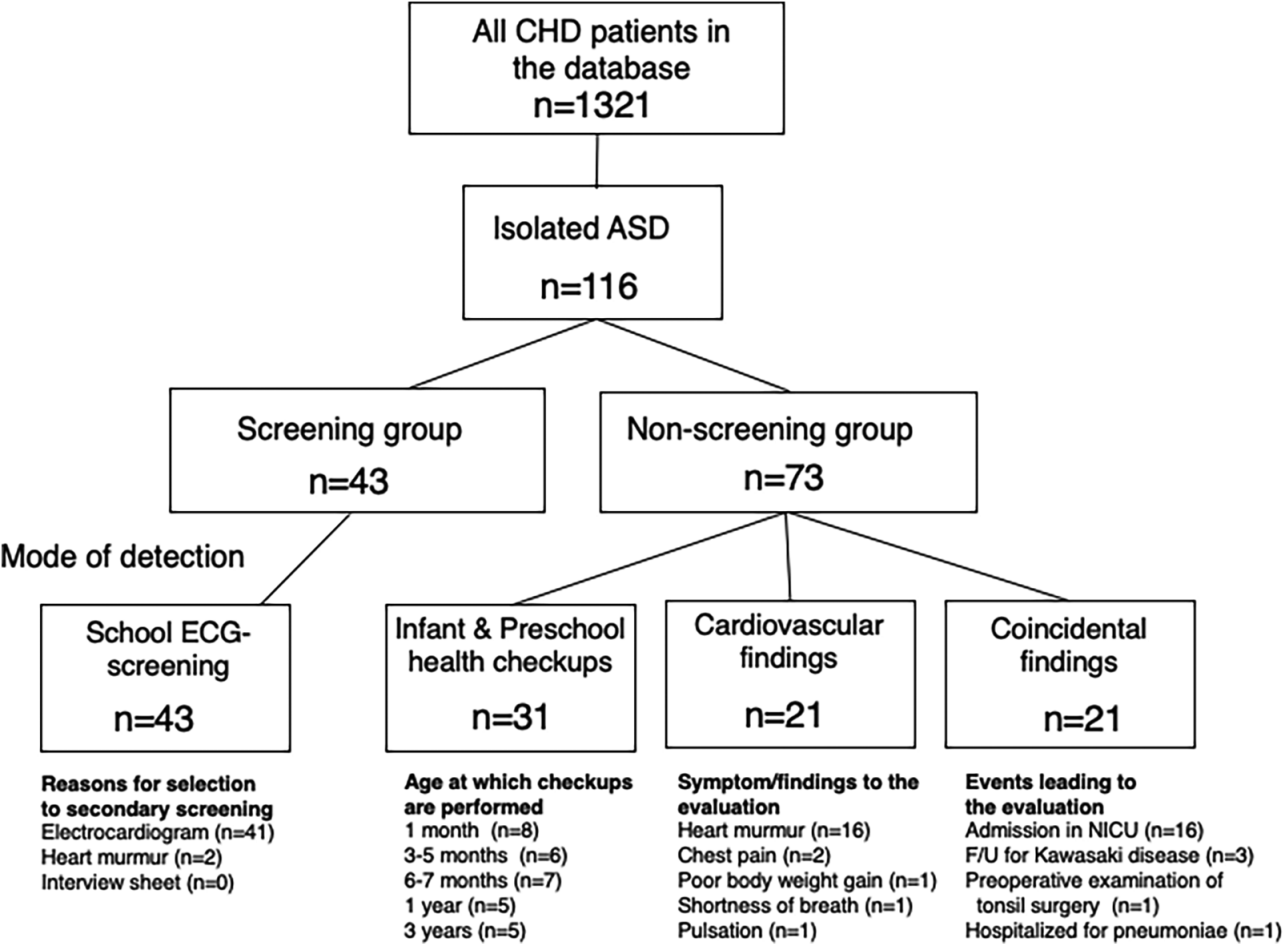
Study population. The patient database (*n* = 1,321) was searched for atrial septal defect (ASD) and patients with isolated atrial ASD examined at the age ≤18 years old, during 2009–2019 in mie university hospital (*n* = 116) were identified. Patients with sinus venosus ASD with partial anomalous pulmonary venous connection or patients with genetic disorders (e.g., down syndrome) were included. Patients were divided into the four groups according to the mode of detection: in school ECG screening; from ASD-related symptom or physical findings in outpatient clinics; in preschool health checkups; as an incidental finding for other reasons. Patients detected by school ECG screening were defined as the screening group and the latter three groups were collectively described as the non-screening group. CHD, congenital heart disease; ASD, atrial septal defect; ECG, electrocardiography; NICU, neonatal intensive care unit; F/U, follow-up.

The patients were subgrouped into four categories according to the mode of detection: (1) school ECG screening, (2) cardiovascular findings related to ASD in outpatient clinics, (3) infant and preschool health checkups, and (4) echocardiogram obtained for other reasons. Patients detected by school ECG screening were described as the Screening group, and the latter three groups were collectively described as the Non-screening group (Table 2 and Figure 1). All parameters collected were compared between the groups.

**Table 2:** Demographic and clinical findings of the patients.

	Overall patients (*n* = 116)	Mode of detection				
		Screening group	Non-screening group			
		School ECG-screening (*n* = 43)	All in non-screening (*n* = 73)			
				Infant & preschool health checkups (*n* = 31)	Cardiovascular findings (*n* = 21)	Coincidental findings (*n* = 21)
Female sex, *n* (%)	63 (54.3)	22 (51.1)	41 (56.1)	20 (64.5)	13 (61.9)	8 (38.1)
Age at diagnosis (year) median (IQR)	3.0 (0.12–6)	7.0 (6–13)	0.41 (0–2)	0.5 (0.08–1)	1.5 (0.17–5.5)	0 (0–0.3)
Age at catheterization (year), median (IQR)	8.9 (5.9–12.8)	11.1 (7.8–14)	7.1 (4.1–11.0)	7.0 (4.1–8.9)	9.9 (5.7–12.5)	5.9 (1.8–10.7)
Type of defect						
Isolated, *n* (%)	114 (98.3)	41 (95.3)	73 (100)	31 (100)	21 (100)	21 (100)
Sinus venosus w/ PAPVC, *n* (%)	2 (1.7)	2 (4.7)	0	0	0	0
Coexisting diseases						
Down syndrome, *n* (%)	8 (6.9)	0	8	0	0	8 (38)
Other genetic disorders, *n* (%)	1 (0.9)	1 (2.3)	0	0	0	0
Treatments						
Catheter closure, *n* (%)	56 (48.2)	24 (55.8)	32 (45.1)	13 (41.9)	9 (42.9)	10 (47.6)
Surgical closure, *n* (%)	57 (49.1)	19 (44.1)	39 (54.9)	18 (58.1)	12 (57.1)	8 (38.1)
Closure not performed	3 (2.2)	0	3 (4.2)	0	0	3 (14.2)
Presence of heart murmur						
At diagnosis, *n* (%), (the number of available data)	59 (58.4) (101)	12 (36.3) (33)	47 (69) (68)	27 (93) (29)	16 (84.2) (19)	4 (20) (20)
At catheterization, *n* (%), (the number of available data)	44 (40.4) (109)	11 (25.6) (43)	33 (50) (66)	19 (63.3) (30)	9 (56.3) (16)	5 (25) (20)

Data are presented as median (interquartile range, IQR). Other categorical data are presented as *n* (%). Percentages were calculated on the basis of the available data in overall or each group. Sinus venosus w/PAPVC, sinus venosus defect with partial anomalous pulmonary venous connection.

ECG, electrocardiography.

### Statistics

Values were presented as numbers and percentages for categorical data and mean ± SD or median and interquartile range for continuous variables as appropriate. Comparisons between the groups were made using the Fisher’s exact test for categorical data, unpaired *t*-test for normally distributed data, and Mann–Whitney *U*-test for variables that were not normally distributed. Normality and Lognormality tests were used to evaluate whether data were normally distributed. Pulmonary and systemic blood flows were calculated with the Fick method with oxygen consumption estimated by the method of LaFarge and Miettinen. The analysis was performed using GraphPad Prism version 9.0. A two-sided *p*-value <0.05 was considered statistically significant.

## Results

A total of 116 patients (53 boys and 63 girls) with ASD were included in the study, in which the median age at diagnosis and at catheterization was 3.0 [interquartile range (IQR), 0.12–6.0] years and 8.9 (IQR, 5.9–12.8) years, respectively. Forty-three patients (37%) were detected through ECG screening (Screening group), while the remaining 73 patients were diagnosed through other means (Non-screening group) (Figure 1). The Non-screening group included 31 patients (27%) diagnosed in infant or preschool health check-ups, 21 patients (18%) diagnosed by cardiovascular findings in outpatient clinics, and 21 patients (18%) diagnosed incidentally during echocardiograms obtained for other reasons. Forty-one (95%) of the 43 patients in the Screening group were picked up by ASD-related ECG abnormalities. The other 2 patients in the Screening group were diagnosed with ASD after a heart murmur was noted during school examinations. In one of them, an ECG at the time of catheterization showed IRBBB. The other demographic and clinical characteristics were shown in Figure 1 and Table 2.

**Figure 2:**
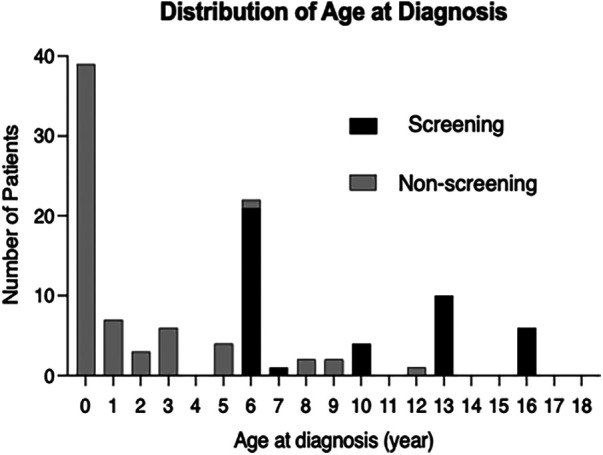
Distribution of the age at diagnosis of atrial septal defect. Distribution of age at diagnosis for overall patients with atrial septal defect (ASD) (*n* = 116). Black boxes indicate the patients with ASD who were detected by the ECG-based screening at school (screening group); gray boxes indicate the patients with ASD which were detected not by the school ECG screening (non-screening group).

The age distribution at diagnosis showed that the largest number of patients were diagnosed before one year of age, and most of patients aged six or older were diagnosed at the age of school ECG screening (Figure 2). Out of a total of 116 patients, 67 (58%) were diagnosed during preschool age and 49 (42%) were diagnosed at six years of age or older. Among the patients diagnosed at six years of age or older, 43 out of 49 (88%) were identified through school ECG screening. In the Non-screening group, only two out of the 6 patients diagnosed at six years of age or older were prompted by heart murmur (Supplementary Table S2). Events prompting cardiovascular examination by age range is shown in Supplementary Table S3.

**Figure 3:**
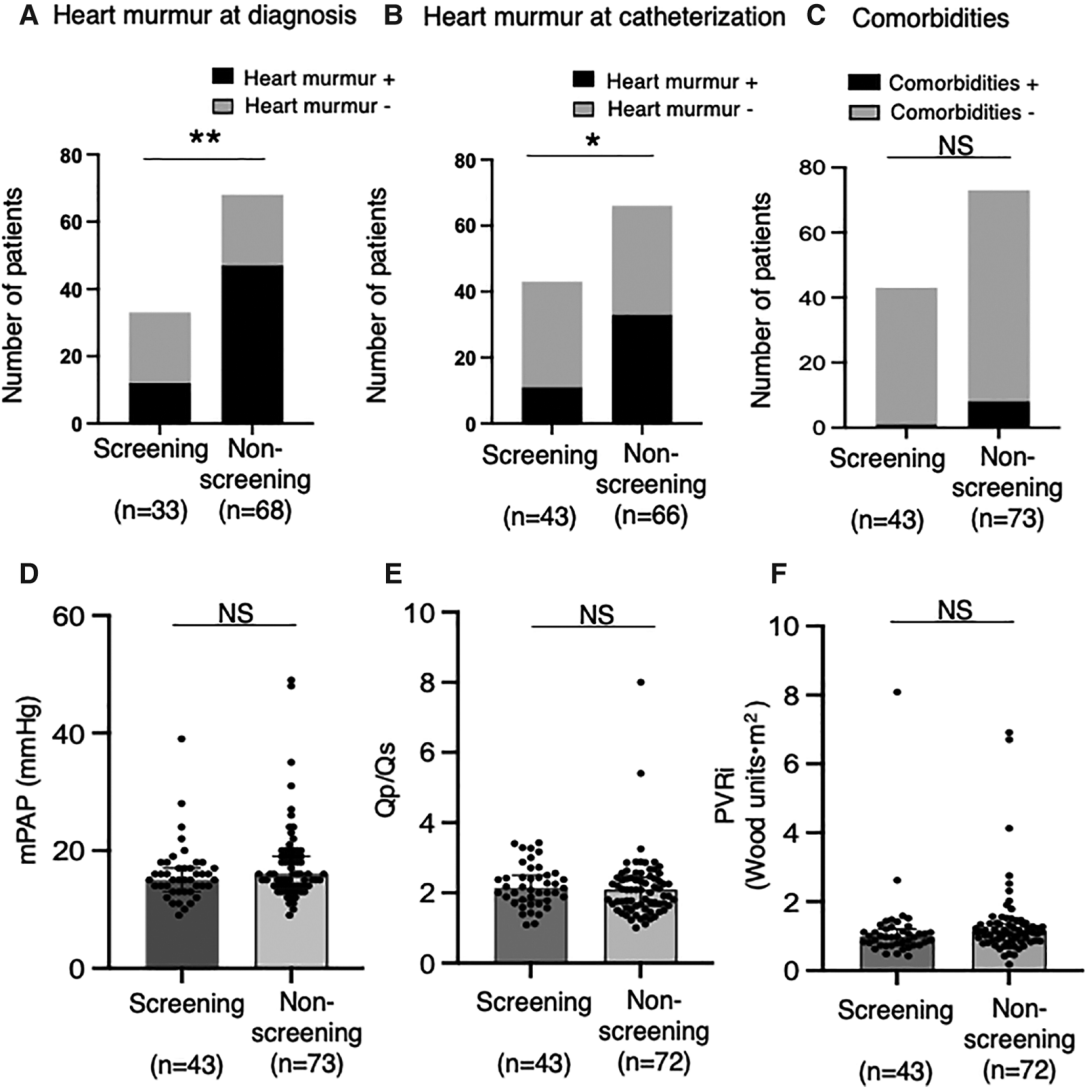
Heart murmur and pulmonary hemodynamic parameters in the screening and non-screening groups. The percentages of patients with heart murmur at diagnosis (**A**) and at catheterization (**B**) was compared between the screening (*n* = 33 at diagnosis, *n* = 43 at catheterization) and the non- screening groups (*n* = 68 at diagnosis, *n* = 66 at catheterization). (**C**) The percentages of patients with comorbidities (i.e., chromosome abnormalities) compared between the screening (*n* = 43) and the non-screening groups (*n* = 73). Mean pulmonary artery pressure (**D**), ratio of pulmonary-to-systemic flow (**E**) and pulmonary vascular resistance index (**F**) assessed by right heart catheterization were compared between the screening (*n* = 43) and the non-screening groups (*n* = 73 for mPAP; *n* = 72 for Qp/Qs and PVRi). Black boxes indicate the patients with atrial septal defect (ASD) who had heart murmur (≥Levine2); gray boxes indicate the patients with ASD who did not have heart murmur (≥Levine2) (**A**,**B**). Comparisons between the groups were made using the Fisher's exact test (**A–C**). Values are shown as individual dots along with median and interquartile ranges (**D**–**F**). Mann-Whitney *U* test was used for analysis. NS, not significant; mPAP, mean pulmonary artery pressure; Qp/Qs, ratio of pulmonary-to-systemic flow; PVRi, pulmonary vascular resistance index. *p < 0.05; **p < 0.01.

**Figure 4:**
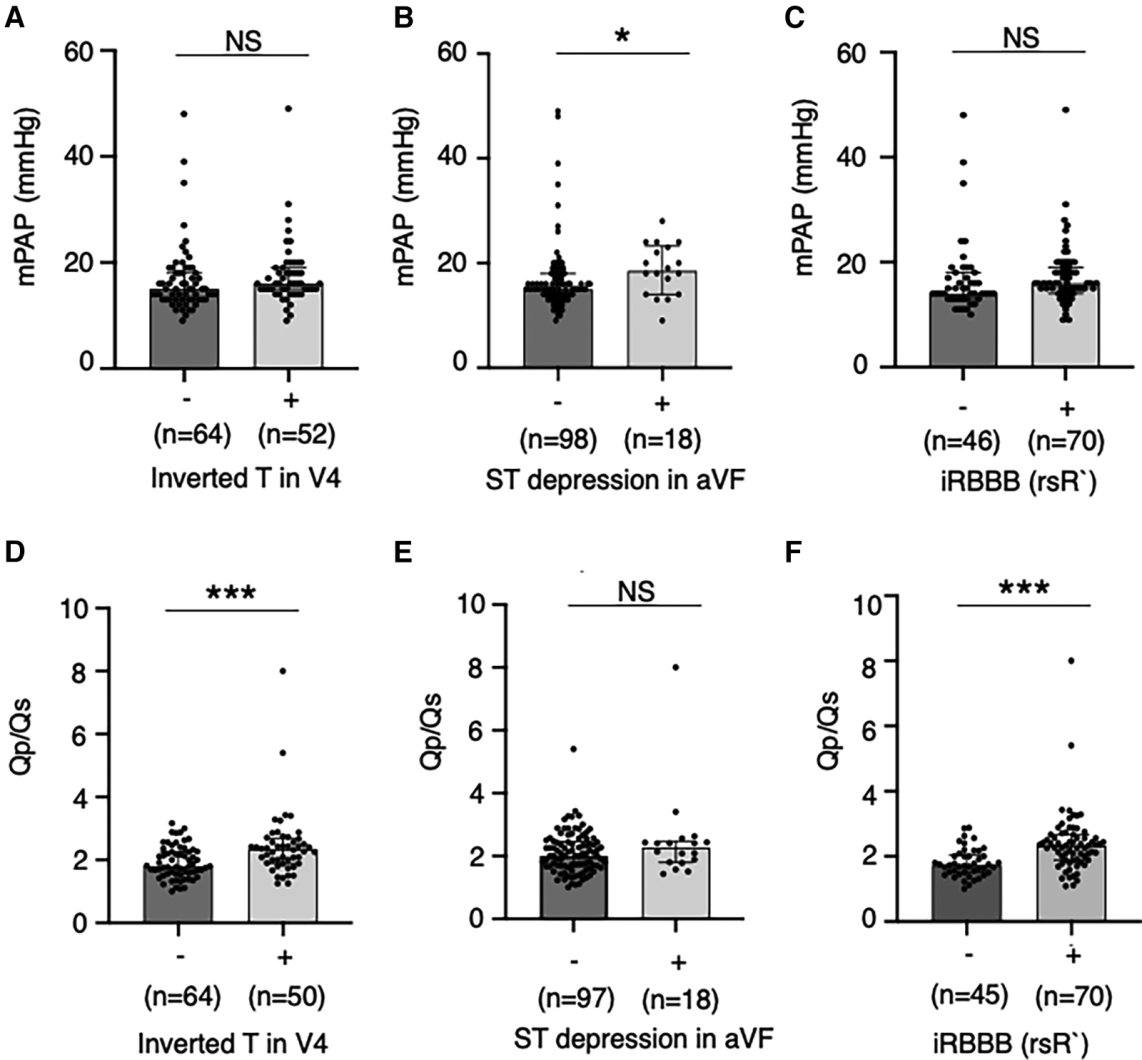
Pulmonary hemodynamic parameters by the abnormal electrocardiographic findings. Mean pulmonary artery pressures (**A–C**) and ratio of pulmonary-to-systemic flow (**D,E**) were compared between patients with and without relevant ECG findings including inverted T in the lead V4, ST depression in the lead aVF and rsR’ type of incomplete right bundle branch brock. Values are presented as individual dots along with median and interquartile range. Mann–Whitney *U*-test was used for analysis. *p < 0.05. ***p < 0.001. NS, not significant; mPAP, mean pulmonary artery pressure; Qp/Qs, ratio of pulmonary-to-systemic flow; RBBB, right bundle branch block.

In overall patients, heart murmur (Levine 2/6 or higher) was found in 58.4% of patients at diagnosis and 40.4% at catheterization. The percentage of patients with significant heart murmurs was lower in the Screening group than in the Non-screening group (36.3% vs. 69.1% at diagnosis, *p* < 0.01, Figure 3A; 25.6% vs. 50% at catheterization, *p* < 0.05, Figure 3B). The percentage of patients with comorbidities (e.g., genetic disorders) tended to be higher in the Non-screening group than in the Screening group, although this was not statistically significant (Figure 3C). The auscultatory findings by age group were shown in Supplementary Table S4. The mean pulmonary artery pressure (mPAP), pulmonary-to-systemic flow ratio (Qp/Qs), and pulmonary vascular resistance index (PVRi) were comparable between the Screening and Non-screening groups in overall patients (Figures 3D–F) and in patients without comorbidities such as Down syndrome (Supplementary Figures S1A–C). The proportion of patients with clinically relevant ECG findings at catheterization, including rsR' type of iRBBB (lead V1 or V2), inverted T in V4 (invTV4) and ST depression in aVF, as well as right axis deviation, iRBBB and crochetage pattern, were comparable in both groups (Table 3). The patients with a composite ECG parameter (rsR' type of IRBBB, invT4 or ST depression in aVF) accounted for 79% in Screening group, which was higher than in Non-screening group (62%, *p* = 0.02) (Table 3). Eight patients (21%) with the negative composite ECG parameter at catheterization in the screening group were prompted by heart murmur (*n* = 1), PAC (*n* = 1), non-rsR’ type of iRBBB (*n* = 4), and 2 patients with negative invTV4 at catheterization but with positive invTV4 at school ECG. Of note, although ECG abnormality was typically rsR' type of iRBBB, some patients had invTV4 or ST depression in aVF without IRBBB (Supplementary Figures 2A,B). These ECG findings were correlated with hemodynamic parameters. Patients with rsR' type of iRBBB had higher Qp/Qs than those without iRBBB (rsR') (2.3 vs. 1.7, *p* < 0.01) and patients with invTV4 had higher Qp/Qs than those without invTV4 (2.3 vs. 1.8, *p* < 0.01) (Figure 4). Patients with ST depression in the lead aVF had higher mPAP (18.5 mmHg vs. 15.0, *p* < 0.02, Figure 4).

**Table 3:** Electrocardiographic findings at catheterization.

	ALL (*n* = 116)	Screening group (*n* = 43)	Non-screening group (*n* = 73)	*p*
(1) iRBBB (rsR’)	70 (60%)	30 (70%)	40 (55%)	0.12
(2) Inverted T in V4	52 (45%)	23 (54%)	29 (40%)	0.18
(3) ST depression in aVF	18 (16%)	8 (19%)	10 (14%)	0.60
(4) iRBBB	89 (77%)	37 (86%)	52 (71%)	0.07
(5) Right axis deviation	22 (19%)	6 (14%)	16 (22%)	0.34
(6) crochetage pattern	62 (53%)	26 (60%)	36 (43%)	0.26
Composite				
ANY ONE of (1–3)	80 (69%)	34 (79%)	46 (63%)	0.02
ANY ONE of (1–6)	103 (89%)	42 (98%)	64 (88%)	0.09

Data are presented as *n* (%). Percentages were calculated on the basis of the data in each group.

The ECG findings were classified into the following three categories according to the guidelines (8).

Category A: Findings requiring secondary screening or further examination.

Category B: Findings not requiring secondary screening (if other abnormal findings are absent).

Category C: Findings not requiring heart disease screening at schools.

Composite (1–3) meet the secondary selection criteria in the JCS guidelines. (Category A).

Composite (4–6) do not meet the criteria. (Category B or C).

iRBBB (rsR’): rsR’ type of incomplete right bundle branch block.

iRBBB: incomplete right bundle branch block.

## Discussion

**Figure 5:**
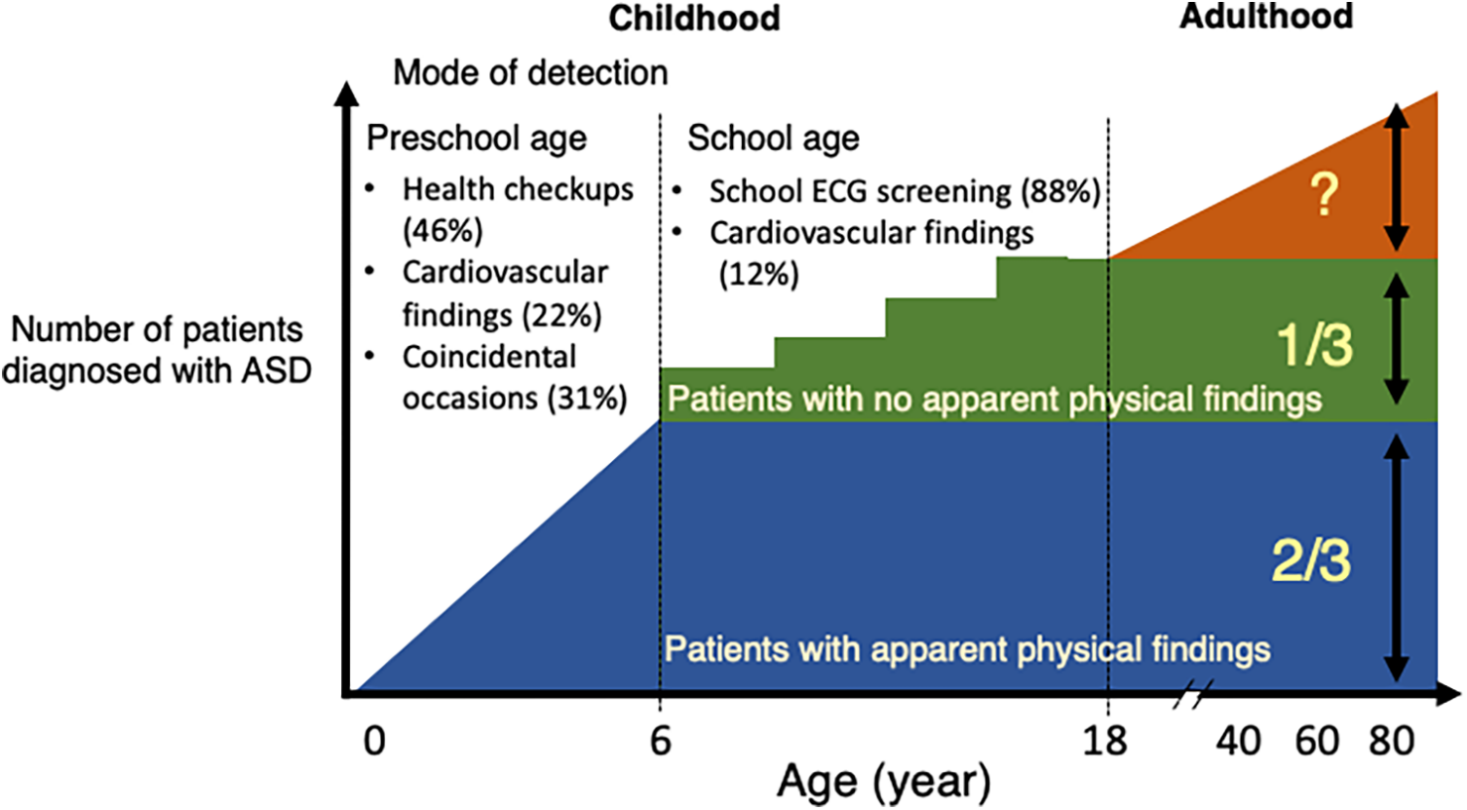
Summary diagram. The proportion of hemodynamically significant atrial septal defect (ASD) patients by the mode of detection in Japan. Approximately two thirds of pediatric patients with ASD were detected during the preschool age through health checkups, in outpatient clinics or as an incidental finding for other reasons. The remaining 1/3, who were mostly murmurless and detected in school ECG screening after 6 years of age. The proportion of undetected ASD patients who may develop comorbidities in adulthood in the present Japanese healthcare system is unknown. ECG, electrocardiogram; ASD, atrial septal defect.

This is the first study to investigate the impact of school ECG screening on the diagnosis of hemodynamically significant ASD in a single tertiary medical service area in Japan. Firstly, the school ECG screening group accounted for 88% of patients diagnosed at school age and 37% of overall pediatric patients in Japan, with the 3 corresponding peaks of the number of patients at diagnosis. Secondly, compared with the non-ECG screening group which were mainly detected by the health care and health checkups in infancy or preschool period, the ECG screening group had a lower proportion of audible heart murmur at catheterization, but exhibited similar levels of hemodynamic parameters. Thirdly, patients who were positive for a composite ECG parameter (the presence of rsR' type of iRBBB, inverted T in V4, or ST depression in the aVF lead) accounted for 79% of the ECG screening group at catheterization, and each of these parameters was correlated with hemodynamic parameters in the overall patient population. The present study suggests that the inclusion of school ECG screening, using the present composite ECG parameter, in the pediatric healthcare system may help detect otherwise undetectable ASD patients with clinically relevant hemodynamics (Figure 5).

The coverage of this study in the tertiary medical service area may be reasonably high considering the estimated incidence of ASD patients. This study was conducted at the only tertiary referral center in a prefecture in Japan, which covers approximately 310,000 of the population under 18 years of age in the prefecture, with an annual number of live births of approximately 10,000 (16). Based on the current study enrollment of 116 pediatric ASD patients over 11 years from 2009 to 2019, the estimated incidence of pediatric ASD patients was 28 per 100,000 live births. Previous reports on ASD diagnosis using echocardiography have reported the incidence ranging from 100 to 390 per 100,000 live births (2, 17, 18), with spontaneous closure rates of 62%–89% (17, 19). Calculating patients with indications for closure from these reports yields 11–148 per 100,000 live births. Despite being a retrospective study, the coverage rate in this study may be reasonably high considering the estimated incidence of ASD patients for shunt closure in the tertiary medical service area.

ECG screening has been performed for all school children in Japan (8) and for individuals participating in competitive sports in parts of Italy (20). Early studies have shown that ASD was included in the list of diseases prompted to be diagnosed by ECG screening in addition to the substrate for sudden cardiac death (8, 14, 15, 21). However, it was unclear how significantly this healthcare system impacts the detection of ASD in the clinical settings. In the present study, the school ECG screening group accounted for 88% of patients diagnosed at school age and 37% of overall pediatric patients in a tertiary medical service area, with the three peaks of the age distribution at diagnosis that coincided with the age of ECG screening. In contrast, a study in Boston identified ASD in school-age patients predominantly through incidental echocardiography or the presence of a heart murmur. In preschool children, however, a similar proportion of detection through heart murmurs, cardiovascular findings, or incidental echocardiography were confirmed in both studies. These findings suggest that the implementation of school ECG screening has an impact on the detection of ASD in overall children and in school students. Furthermore, in the present study, compared with the non-screening group, the screening group exhibited a lower proportion of audible heart murmur despite comparable levels of hemodynamic parameters. These findings suggest that ECG screening program could be an effective strategy for detecting hemodynamically significant ASD in students, who are asymptomatic and murmurless.

In this study, the prevalence of a composite ECG parameter [the presence of iRBBB (rsR'), invTV4, or ST depression of aVF] was 63% in the non-screening group as well as 79% in the screening group, suggesting a reasonably high sensitivity of this parameter for detecting hemodynamically significant ASD. The sensitivity of rsR' QRS pattern in detecting hemodynamically significant ASD (55% in the non-screening group and 70% in the screening group) was consistent with previous reports (54%–79%) (2, 15, 18, 22). Although the ECG finding invTV4 in ASD patients was reported previously (10, 23), the sensitivity of invTV4 or ST depression in aVF in detecting ASD was unknown. These findings suggest that the present composite parameter may help in detecting ASD. Furthermore, these three parameters had hemodynamic relevance. The present study also showed that iRBBB (rsR') that has been regarded as reflecting right ventricular overload and invTV4 were associated with higher Qp/Qs, which is consistent with the previous studies (18, 23, 24). Patients with ST depression in aVF, an ECG index suggestive of right ventricular pressure load (25), was associated with the higher mPAP, not Qp/Qs. Furtheremore, these parameters have high specificity: the prevalence of iRBBB (rsR') and invTV4 were reported in general Japanese school children (IRBBB: 0.983% in male and 0.410% in female; InvTV4: 0.12% in male and 0.15% in female in 1st grader, 0.10% in male and 0.13% in female in the 7th graders, 0% in male and 0.04% in female in the 10th graders) (26, 27).

In addition to the present three ECG parameters, we also evaluated right-axis deviation and “crochetage” pattern in the present study. Although these parameters increased the sensitivity in detecting ASD, the specificity of such parameters was reported to be low. The sensitivity and specificity of “crochetage” pattern for ASD detection has been shown to be lower in children than in adults (sensitivity: 31.7 vs. 57; specificity: 86 vs. 92) (28, 29), and a relatively high prevalence (2.74%) has been reported in children with normal echocardiographic findings (30). Collectively, the proposed composite ECG parameter may be useful for the screening of hemodynamically significant ASD in this age group.

## Limitations

There are four limitations in this study. Firstly, although patients were enrolled using a prospectively constructed patient database, the retrospective nature of this study should be considered in interpreting the results. Auscultatory findings are a subjective indicator to promote the diagnosis of ASD. Although board-certified pediatric cardiologists perform auscultation at the time of catheterization, there may be inter-observer variability. Secondly, the specificity or the false positive rate of the ECG index for ASD diagnosis was not examined in the present study or in the literature, although the low prevalence of relevant ECG parameters, including 0.41%–0.98% for iRBBB (rsR') and 0.04%–0.15% for invTV4, were reported in general Japanese school children (8, 26, 27). Thirdly, the limitations of the school ECG screening in extracting ASD: the present composite parameter was positive in only 79% of patients. In addition, given the natural history of ASD during life, patients who did not have significant shunting in childhood may have increased shunt blood flow in adulthood, due to age-related changes in cardiac function (i.e., decreased left ventricular compliance). Fourth, cost-effectiveness cannot be determined in this study. Tanaka et al. proved the cost-effectiveness of school ECG screening from the viewpoint of sudden death prevention (31). Since the detection of ASD is a byproduct of this program, it may be cost-effective as well. In the future, automated ECG recording and application of deep learning systems to ECG interpretation may further improve cost-effectiveness.

## Implications

Firstly, patients treated for otherwise undetectable ASD, who were prompted by the present ECG screening program, may be free from comorbidities associated in adulthood. Secondly, on top of the detection of substrates for sudden cardiac death, ASD should be included in the target heart diseases for pediatric ECG screening. Thirdly, the present composite parameter (presence of rsR' type of iRBBB, inverted T in V4 or ST depression) may be recommended for the ECG screening of ASD.

## Conclusions

The present study shows that school ECG screening detects otherwise unrecognized ASD, which prompted the diagnosis of the majority of patients at school age and >one-third of overall patients in Japan. These findings suggest that ECG screening program could be an effective strategy for detecting hemodynamically significant ASD in students, who are asymptomatic and murmurless.

## Data Availability

The original contributions presented in the study are included in the article/**Supplementary Material**, further inquiries can be directed to the corresponding authors.
