# Supplementary material for: School electrocardiography screening program prompts the detection of otherwise unrecognized atrial septal defect in children in Japan
Source: Front Pediatr. 2024 Jun 3;12:1396853. doi: 10.3389/fped.2024.1396853 (PMC11180781; doi:10.3389/fped.2024.1396853)
Supplement: Supplementary file 1 [file Datasheet1.pdf]

Supplementary Material

School electrocardiography screening program prompts the detection of otherwise unrecognized atrial septal defect in children in Japan.

Noriko Yodoya MD, Hirofumi Sawada MD PhD, Yoshihide Mitani MD PhD, Hiroyuki Ohashi MD, Naoki Tsuboya MD, Kazunobu Ohya MD, Mami Takeoka MD, Hidetoshi Hayakawa MD PhD and Masahiro Hirayama MD PhD

Table of contents

Supplemental Table 1 Summary of ECG criteria for secondary screening related to atrial septal defect in school-based ECG screening in Japan

Supplemental Table 2 Symptoms or findings leading to the evaluation in patients in the non-screening group diagnosed at 6 years of age or older

Supplemental Table 3 Events prompting cardiovascular examination

Supplemental Table 4 Auscultatory characteristics by age at diagnosis and catheterization

Supplemental Figure 1 Pulmonary hemodynamic parameters assessed by catheterization in patients after excluding syndromic patients

Supplemental Figure 2 Representative ECG findings in atrial septal defect

Supplemental Table 1

Summary of ECG criteria for secondary screening related to atrial septal defect in school-based ECG screening in Japan

1. Incomplete right bundle branch block
   Incomplete right bundle branch block (rsR'): QRS width <0.12 sec (middle and high school), < 0.10 sec (lower elementary school) and R'>R (V1 or V2) and R'V1≥|SV1|
   Incomplete right bundle branch block other than rsR' type are not extracted for secondary screening.
2. T wave inversion
   T wave is negative or biphasic and negative area ≥ 0.1 mV (I, II, aVL [R ≥ 0.5 mV], aVF, any of V4 to V6 for elementary school students or any of V3 to V6 for middle or high school students.
3. ST segment depression
   ST segment depression ≥ 0.05 mV and ST segment is horizontal or descending (I, II, aVL, aVF, V1 to V6)
4. Right axis deviation
   Right axis deviation defined as QRS axis ≥ +120 degree alone are not extracted for secondary screening.
5. “crochetage” pattern
   The “crochetage” pattern is not included in the criteria.

The ECG findings were classified into the following three categories according to the guidelines. [1]

Category A: Findings requiring secondary screening or further examination.

Category B: Findings not requiring secondary screening (if other abnormal findings are absent).

Category C: Findings not requiring heart disease screening at schools.

The ECG findings, 1. Incomplete right bundle branch block (rsR'), 2. T wave inversion or 3. ST segment depression, were classified as Category A. Right axis deviation was classified as Category B. The “crochetage” pattern is not included in the guideline. Therefore, subjects with ANY ONE of 1 or 2 or 3. (Incomplete right bundle branch block (rsR'), T wave inversion or ST segment depression) meet the criteria for the secondary screening. Subjects with right axis deviation do not meet the criteria for the secondary screening, if other abnormal findings are absent.

Reference

1) Sumitomo N, Baba R, Doi S, Higaki T, Horigome H, Ichida F, Ishikawa H, Iwamoto M, Izumida N, Kasamaki Y, et al. Guidelines for Heart Disease Screening in Schools (JCS 2016/JSPCCS 2016)- Digest Version. *Circ J.* 2018; 82: 2385-2444. doi: 10.1253/circj. CJ-66-0153

Supplemental Table 2. Symptoms or findings leading to the evaluation in patients in the non-screening group diagnosed at 6 years of age or older.

**Symptoms or findings**	**n**
Heart murmur	2
Chest pain	1
Shortness of breath	1
Pulsation	1
Preoperative examination for tonsillectomy	1

Supplemental Table3. Events prompting cardiovascular examination.

Age at Diagnosis (year)	School ECG (n=43)	Infant & Preschool Health checkups (n=31)	Cardiovascular findings (n=21)	Coincidental findings (n=21)
<1	NA	21	9	17
1-5	NA	10	7	3
6-10	27	NA	5	1
11-18	16	NA	0	0

NA：not applicable

ECG: Electrocardiography

Supplemental Table 4. Auscultatory characteristics by age at diagnosis and catheterization

Presence of heart murmur at diagnosis				
Age at diagnosis, year	<1<br>(n=47)	1-5<br>(n=20)	6-10<br>(n=32)	11-18<br>(n=17)
the number of available data	45	19	25	12
Patients with heart murmur (Levine≧2), n (%)	29 (64)	15 (79)	8 (32)	7 (38)
Presence of heart murmur at catheterization				
Age at catheterization, year	<1<br>(n=3)	1-5<br>(n=27)	6-10<br>(n=46)	11-18<br>(n=40)
the number of available data	3	25	44	37
Patients with heart murmur (Levine≧2), n (%)	2 (67)	17 (68)	16 (36)	9 (24)

Data are presented as n (%). Percentages were calculated on the basis of the data in each group.

Supplemental Figure 1

Pulmonary hemodynamic parameters assessed by catheterization in patients after excluding syndromic patients.

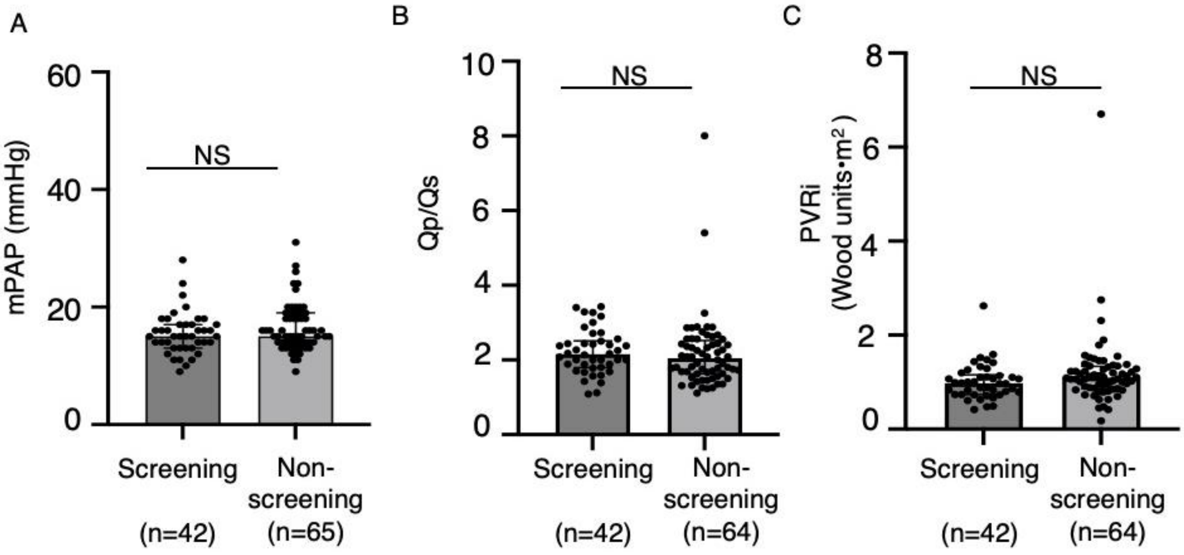

Mean pulmonary artery pressure (mPAP, A), ratio of pulmonary-to-systemic flow (Qp/Qs, B) and pulmonary vascular resistance index (PVRi, C) assessed by right heart catheterization were compared between the screening (n=42) and the non-screening groups (n=65 for mPAP; n=64 for Qp/Qs and PVRi) after excluding the patients with syndromic disorders (e.g. Down syndrome). Values are shown as individual dots along with median and interquartile range. Mann-Whitney *U* test was used for analysis.

ASD: atrial septal defect; ECG: electrocardiography; NS: not significant; mPAP: mean pulmonary artery pressure; Qp/Qs: Ratio of pulmonary-to-systemic flow; PVRi: Pulmonary vascular resistance index.

Supplemental Figure 2

Representative ECG findings in atrial septal defect

A. Isolated inverted T-wave in V4 lead without rsR' type iRBBB (6-year-old male)

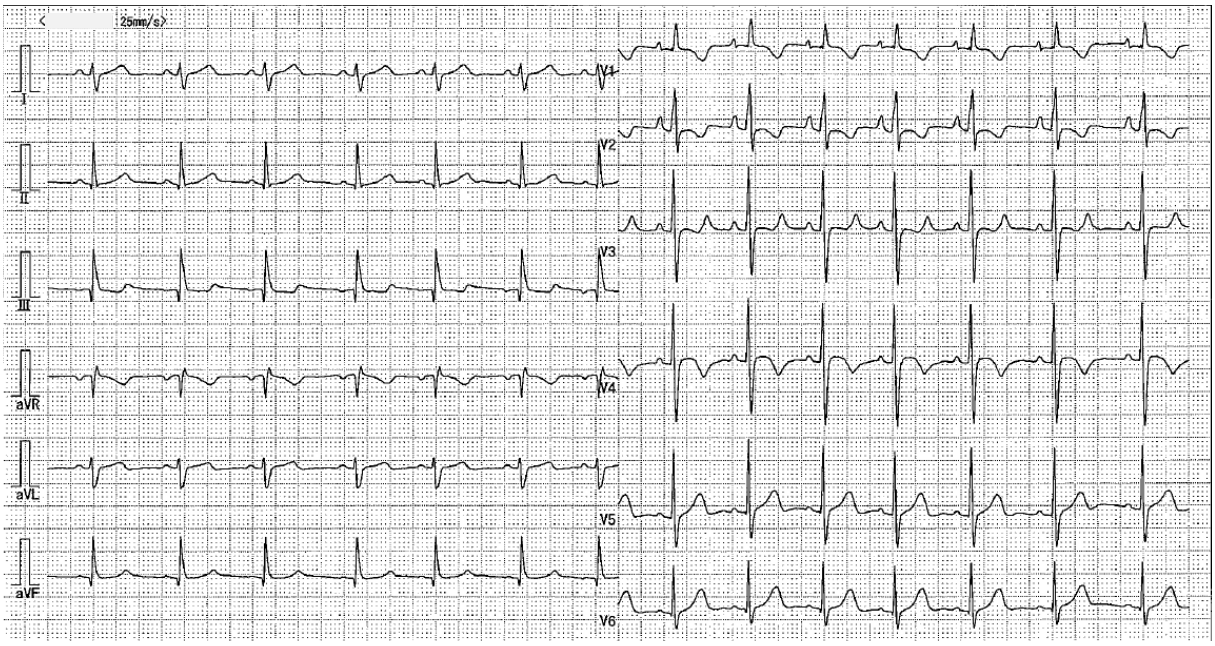

B. ST depression in aVF without rsR' type iRBBB (6-year-old female)

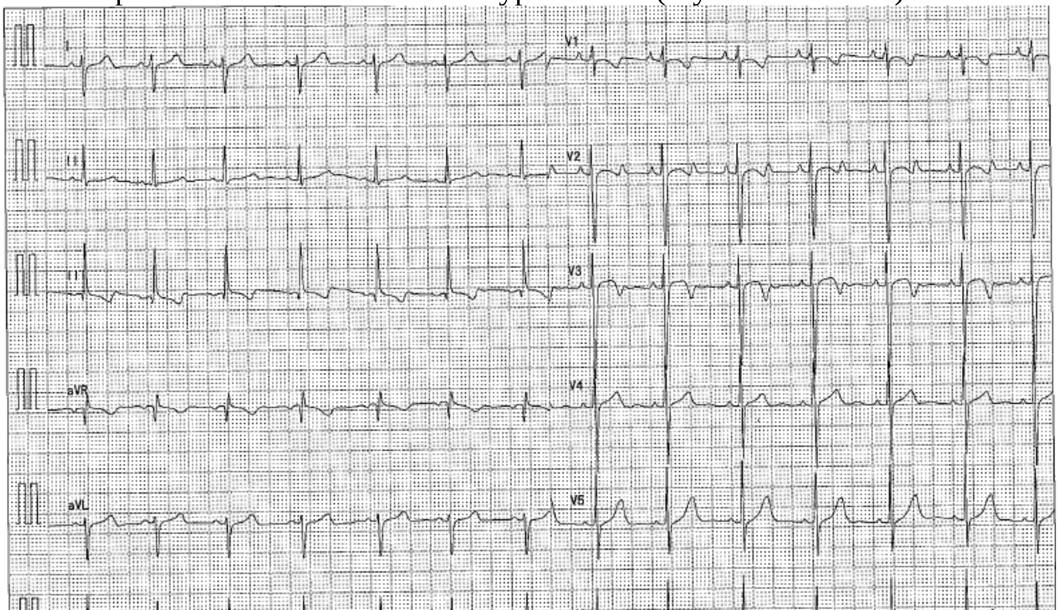

Electrocardiograms showing isolated inverted T-wave in V4 (A) and ST depression in aVF lead (B) without rsR' type iRBBB recorded in patients with atrial septal defect.

RBBB: right bundle branch block.
